# Maintaining Drosha expression with Cdk5 inhibitors as a potential therapeutic strategy for early intervention after TBI

**DOI:** 10.1038/s12276-023-01152-4

**Published:** 2024-01-10

**Authors:** Lu Huang, Li Xia, Tiejian Nie, Bozhou Cui, Jianjun Lu, Fangfang Lu, Feiyan Fan, Dongni Ren, Yuan Lu, Guodong Gao, Qian Yang

**Affiliations:** 1 Department of Experimental Surgery, Tangdu Hospital, The Fourth Military Medical University, Xi’an, 710038 Shaanxi China; 2 Department of Anesthesiology, Tangdu Hospital, The Fourth Military Medical University, Xi’an, 710038 Shaanxi China; 3 Department of Neurosurgery, Tangdu Hospital, The Fourth Military Medical University, Xi’an, 710038 Shaanxi China

**Keywords:** Cell death in the nervous system, Trauma

## Abstract

Traumatic brain injury (TBI) is a major cause of death and disability in adults. The pathological process of TBI involves a multifactorial cascade in which kinases have been proven contribute to interactions between relevant factors and amplification of signaling cascades. Cyclin-dependent kinase 5 (Cdk5) is a promising kinase that has been implicated in various brain disorders, including TBI. However, the mechanism by which Cdk5 induces neuronal damage remains unclear. Here, we show for the first time that Drosha, a key enzyme in microRNA biogenesis, is a pivotal substrate of abnormally activated Cdk5. Cdk5-mediated phosphorylation decreases Drosha expression and exacerbates nerve injury in TBI. We proved that maintaining Drosha expression via the administration of repurposed Cdk5 inhibitors that were previously studied in clinical trials is a promising approach for the early treatment of TBI. Together, our work identifies Drosha as a novel target for neuroprotective strategies after TBI and suggests Cdk5-mediated regulation of Drosha expression as a potential therapeutic strategy for early TBI intervention.

## Repurposed Cdk5 Inhibitors: A Promising Strategy for TBI Treatment

Traumatic brain injury is a leading cause of death and disability in adults, with secondary injuries often causing more damage than the initial trauma. However, the complex series of events leading to these additional injuries is not well understood. A study by Huang et al. found that a protein called Drosha, which is crucial for the biogenesis of microRNA, is decreased in the injuried brain following TBI. This decrease is due to an kinase called Cdk5, which becomes abnormally active after TBI and phosphorylates Drosha, causing it to degrade. By maintaining Drosha level via Cdk5 inhibitors, the researchers were able to reduce brain damage and improve neurological function of TBI mice. This suggests that stabilizing Drosha levels by repurposed Cdk5 inhibitors could be a promising strategy for early intervention in TBI.

This summary was initially drafted using artificial intelligence, then revised and fact-checked by the author.

## Introduction

Traumatic brain injury (TBI) is a major cause of death and disability in adults. The pathogenesis of TBI involves a complicated series of events that are often divided into primary and secondary injury^1^. Primary injury includes irreversible cellular death at the moment of mechanical impact and leads to the development of secondary injury, which includes cellular hyperexcitability, vasogenic and cytotoxic edema, hypoxia-ischemia, oxidative stress and inflammation, all of which cause the expansion of the primary lesion^2^. Secondary injury may occur within minutes or years after trauma and is considered a possible target for treatment^3^. Studies have shown that the development of secondary injury is caused by a sequential multifactorial cascade that is promoted by critical kinases that mediate crucial protein modifications in the early stage^4^. Kinases (e.g., MAP kinase, ERK 1 and 2, p38 kinase, JNK) have been reported to play specific roles in neurotoxicity, oxidative stress, and inflammation, and modulating kinase activity has shown great promise as a neuroprotective strategy for early intervention after brain trauma^5,6^.

Cyclin-dependent kinase 5 (Cdk5), a unique member of the cyclin-dependent kinase (CDK) family, is critical for neuronal survival and was reported to be activated shortly after brain trauma^7^. The function of Cdk5 is tightly regulated by the interaction of Cdk5 with its regulator, either p35 or p39^8^. After binding to p35, Cdk5 is activated and participates in neuronal development and axonal and dendritic growth^9^. Under stress conditions, p35 and p39 are cleaved to form p25 and p29, respectively, by calpain in the presence of excess calcium in the cytoplasm^10^. The interaction between Cdk5 and p25 is more stable, and the contribution of the Cdk5-p25 interaction to cell death has been demonstrated in neurological emergencies and neurodegeneration^11^. Multiple synthetic inhibitors that affect Cdk5 activity (such as roscovitine, olomoleucine, and purvalanol-A) have been discovered and proven to exert neuroprotective effects in preclinical research on stroke and TBI^12^. The unique substrates of Cdk5 and how this protein is regulated need to be further studied to expand the clinical application of these inhibitors.

In this study, we show for the first time that Drosha, a key enzyme in the biogenesis of microRNA, is a pivotal substrate target of Cdk5. Drosha is crucial for miRNA biogenesis, which involves tightly regulated sequential steps. Together with its cofactor DiGeorge syndrome critical region gene 8 (DGCR8), Drosha cleaves primary miRNAs (pri-miRNAs) into precursor miRNAs (pre-miRNAs), which are further processed to form mature miRNAs in the cytoplasm^13^. Several groups have verified that miRNA expression changes in injured tissues after TBI and that the majority of miRNAs that exhibit expression changes participate in the pathophysiological cascades of secondary injury after TBI^14^. In addition to its classical role in miRNA biogenesis, Drosha participates in the determination of cellular fate via its miRNA-independent functions, as it participates in the DNA damage response^15^, pre-miRNA-like exon alternative splicing^16^, and gene expression regulation^17^ and is also reported to participate in neuronal injury. However, changes in Drosha expression and its role in nerve injury after brain trauma remain unclear. Here, we proved that Drosha was directly phosphorylated by abnormally activated Cdk5 in the injured cortex of CCI model mice, resulting in a decrease in Drosha expression and the exacerbation of nerve injury and neurological deficits. We verified that Drosha overexpression exerted neuroprotective effects in CCI model mice. Considering the abundance of CDK inhibitors and the characteristics of TBI, we identified the time frame during which stabilizing Drosha levels with two promising CDK inhibitors, which have entered clinical trials for cancer treatment, is effective for the early treatment of TBI. In summary, our findings identify Drosha as a novel target for neuroprotective strategies after TBI and reveal the clinical potential of Cdk5-mediated Drosha regulation as a neuroprotective strategy at early time points after TBI.

## Materials and methods

### Animals

All the animal procedures were approved by the Institutional Ethics Committee of the Fourth Military Medical University and were performed in compliance with the ARRIVE guidelines; the experiments were carried out in accordance with the National Institutes of Health Guide for the Care and Use of Laboratory Animals (NIH Publications No. 8023, revised 1978). C57BL/6 J mice were obtained from the Experimental Animal Center of the Fourth Military Medical University, and transgenic mice were obtained from Shanghai Model Organisms Center, Inc (Shanghai, China). The mice were housed under a 12-hour light/12-hour dark cycle under pathogen-free conditions with free access to dry food and water for 1 week before the experiment.

### Controlled cortical impact model

Before the model was established, mice were selected based on weight (19-21 g), randomly numbered and assigned to groups. All the surgical procedures were performed as previously described^18^. Briefly, the animals were anesthetized with 0.15 ml of a 10:1 mixture of ketamine (100 mg/ml) and xylazine (20 mg/ml) and placed in the stereotactic frame of an injury device (68099II, RWD Life Science Co.). The body temperature of the mice was maintained at 37 °C using an isothermal pad. A 5 × 5 mm craniotomy was performed lateral to the central fissure on the left skull centered between lambda and bregma without disrupting the dura. In the injury groups, the surface of the brain was struck with an impactor that had a 2-mm stainless steel tip; the injury was unilateral, the velocity of the impactor was 2 m/s, the depth was set to 1.5 mm, and the dwell time was 0.2 s. After the injury, the bone flaps were replaced and sealed with liquid adhesive (Loctite 454, Henkel Technologies), and the incisions were closed with surgical staples.

### Drug administration

Mice in the different groups received an intraperitoneal injection of vehicle (10% DMSO, 40% PEG300, 5% Tween-80 and 45% saline), roscovitine (40 mg/kg; dissolved in vehicle according to the user guide) or dinaciclib (15 mg/kg, dissolved in vehicle according to the user guide) at the specified time point after controlled cortical impact (CCI). For the treatment of cultured cells, roscovitine and dinaciclib were dissolved in DMSO.

### Viral infection

Ad-MCMV-GFP-Drosha-mt5-FLAG, Ad-MCMV-GFP-Drosha-wt-FLAG, pAAV-CAMKIIa-GFP-2A-Cre, pAAV-CAMKIIa-Drosha shRNA and their negative controls were purchased from Genecham Biotechnology. In the animal experiments, 1 μl of Ad viral particles (9 ×10^10^ pfu/ml) or 0.5 μl of AAV viral particles (8 ×10^12^ pfu/ml) were stereotaxically injected into the primary motor cortex (0.8 ML, 0.0 AP, 1.5 and 1.2 DV). For the infection of cultured cortical neurons, Ad viral particles (MOI = 30) or AAV viral particles (MOI = 20) were administered 3 days after plating.

### Western blotting

Western blotting was conducted as previously described^19^. Primary antibodies against the following proteins/peptides were used: Drosha (1:500, 3410 S, CST), DGCR8 (1:1000, ab191875, Abcam), Bax (1:1000, 2772 S, CST), Cdk5 (1:1000, 14145 S, CST), p35/p25 (1:1000, 2680 S, CST), Caspase 3 (1:1000, 9662 S, CST), cleaved Caspase 3 (1:1000, 9661 S, CST), p-Tau (1:800, 9632 S, CST), α-p/s-substrate (1:1000, 9477 S, CST), β-actin (1:5000, AC026, ABclonal) and FLAG (1:1000, 70586SF, CST).

### Histopathological analysis

For stereology, brains were fixed in 4% paraformaldehyde for 24 h and then cut into serial coronal sections (100 μm) with an Oscillating Vibroslice Motorized Advance Tissue Slicer as previously described^20^. For Nissl staining, brains were fixed in formaldehyde and washed in PBS three times. Then, serial coronal sections (30 μm) were incubated with Nissl staining solution (C0117, Beyotime Biotechnology) for 10 min at 50 °C according to the manufacturer’s instructions.

### Behavioral analyses

An observer who was blinded to the experimental conditions and treatments used the modified neurological severity score (mNSS) to assess motor function, sensory function, reflexes, and balance as previously reported^21^. The wire grip and motion test (WGMT) was performed to evaluate motor function and muscle strength as previously reported^22^. Briefly, a mouse was placed on a thin and horizontal metal wire (45 cm long) suspended (45 cm above a foam pad) between two poles, and the ability of the mouse to grip and pull the wire was evaluated. The test was performed in triplicate, and an average value was calculated for each mouse. The foot-fault test was performed as previously reported with modifications^23^. Briefly, a mouse was placed on a grid with 1-cm spaces between 0.3-cm-diameter metal rods and observed for 2 min. A foot fault was recorded if the paw of the mouse fell through or slipped between the wires during a weight-bearing step. The percentage of foot faults by the right limb relative to the total steps was calculated.

To evaluate the effect of CCI on motor function, the open-field test was conducted 3 days post-TBI as previously reported with modifications^24^. Briefly, a mouse was placed inside a 50 cm by 50 cm by 38 cm activity chamber (San Diego Instruments) for 10 min. After each recording, the chamber was cleaned with 70% ethanol to remove any odor cues. EthoVision XT 8.5 was used to analyze the distance traveled and the velocity.

All the behavioral data were evaluated by an observer who was blinded to the experimental conditions and treatments.

### Cell culture

HEK-293T cells were obtained from American Type Culture Collection and were cultured in Dulbecco’s modified Eagle’s medium (10-013-CVR, Corning) supplemented with 10% fetal bovine serum (16000-044, Gibco) and 100 U/ml penicillin‒streptomycin (15140-122, Invitrogen).

Primary cortical neurons were isolated and cultured as described previously^25^. Briefly, cortical neurons were harvested from C57BL/6 J mice on embryonic days (E)15–17. The neurons were maintained in neurobasal medium (21103-049, Invitrogen) supplemented with B27 (17504-044, Invitrogen), 0.5 mM L-glutamine (25030-081, Gibco), and penicillin‒streptomycin. Primary cortical neurons were plated in 60-mm cell culture dishes at a density of 1 × 10^7^ cells/dish. The cells were cultured at 37 °C in a humidified atmosphere containing 5% CO_2_.

### Plasmid preparation

Plasmids carrying wild-type and mutant Drosha sequences were generated using a Q5 site-directed mutagenesis kit from New England Biolabs (Massachusetts, USA) according to the manufacturer’s instructions. The sequences of the primers are shown in Supplementary Table 1. The sequences in the plasmids were validated by DNA sequencing.

### Immunoprecipitation

Immunoprecipitation was conducted as described previously^26^. Briefly, tissues or cells were lysed in RIPA buffer (P0013C, Beyotime) containing protease cocktail inhibitor (539134, Millipore) and phosphatase inhibitor (524625, Millipore). The samples were incubated with an antibody against Drosha, Cdk5 or FLAG and with protein A/G Sepharose (LSKMAGAG, Millipore).

### In vitro kinase assay

FLAG-tagged wild-type or mutant Drosha was transfected into HEK-293T cells as described previously^25^. The recombinant active Cdk5/p25 protein was purchased from Millipore Sigma (14-477). The Cdk5 kinase assay was performed as previously described with modifications^27^. Briefly, for the in vitro kinase assay, 6.4 μl of 5× kinase reaction buffer, 7.2 μl of ddH_2_O, 0.16 μl of phosphatase inhibitors, 2 μl of 10 mM ATP (A2383, Sigma), and 10 μl of 0.1 μg/μl purified FLAG-tagged proteins were incubated with 5 μl of 0.1 μg/μl Cdk5/p25 at 30 °C for 30 min. To stop the reaction, 5× loading buffer was added to the tube, and the sample was boiled at 100 °C for 5 min.

### TUNEL staining

Terminal deoxynucleotidyl transferase-mediated dUTP nick end labeling staining was performed with a FragEL™ DNA Fragmentation Detection Kit (QIA39, Sigma) according to the manufacturer’s instructions. The TUNEL-positive cells were counted using ImageJ software.

### miRNA sequencing and analysis

Total RNA was extracted using the TRK1002U Kit (LC Sciences, Houston, USA) according to the manufacturer’s protocol. The quantity and purity of the total RNA were analyzed using a Bioanalyzer 2100 and RNA 6000 Nano Kit (Agilent, CA, USA). Approximately 1 µg of total RNA was used to prepare a small RNA library with the TruSeq Small RNA Sample Prep Kit (Illumina, San Diego, USA) according to the manufacturer’s protocol. Then, we performed single-end sequencing (1 × 50 bp) on an Illumina HiSeq 2500 at LC-BIO (Hangzhou, China) following the manufacturer’s instructions. miRNA differential expression based on normalized deep-sequencing counts was analyzed by selectively using Fisher’s exact test, Chi-squared 2 × 2 test, Chi-squared NxN test, Student’s *t* test, or ANOVA. Highly expressed miRNAs were analyzed following the instructions, and the significance threshold was set to 0.05 in the test. Target gene prediction was analyzed by TargetScan 5.0 and miRanda 3.3a software.

### Statistical analysis

Statistical analysis was performed using GraphPad version 8 software. Student’s *t* test was used to analyze differences between two groups, and one-way ANOVA with Dunnett’s post hoc test was carried out analyze differences among multiple groups. The data are expressed as the means ± SEMs. P ≤ 0.05 was considered to indicate statistical significance.

## Results

### Decreased Drosha expression is associated with nerve injury in TBI

**Fig. 1:**
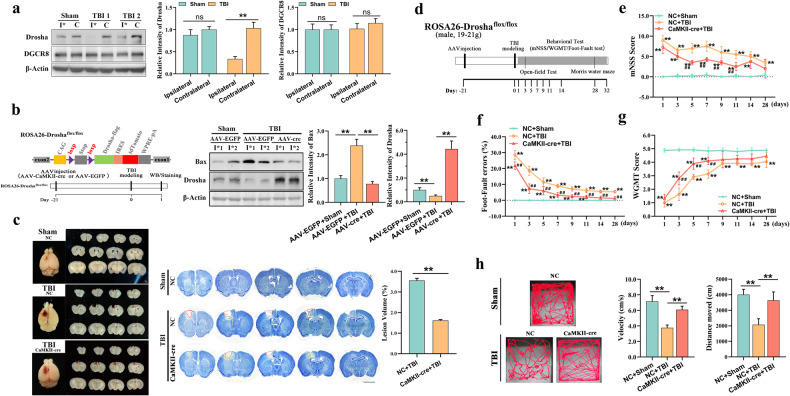
Decreased Drosha expression is associated with nerve injury after TBI. **a** Representative WB showing the protein levels of Drosha and DGCR8 in the injured (I*) and corresponding (C) cortices of CCI model mice (*n* = 4, **P < 0.01). **b** Left: Schematic showing the protocol used to generate transgenic mice overexpressing Drosha and the experimental timelines. Middle: WB showing the change in the expression of the apoptotic protein Bax in the injured cortices of CCI model mice with or without Drosha overexpression (numbers 1 and 2 represent the different mice). Right: Statistical analysis of the relative protein levels of Drosha and Bax in the injured cortices of CCI model mice (*n* = 4, **P < 0.01). **c** Left: Morphological analysis of tissue defects in CCI model mice with or without Drosha overexpression. Middle: Images of Nissl staining showing tissue lesions in CCI model mice with or without Drosha overexpression; Right: Statistical analysis of the cortical lesion volume in CCI model mice with or without Drosha overexpression (*n* = 4, **P < 0.01, bar = 4 mm). **d** Schematic showing the timeline for AAV injection and behavioral testing of CCI model mice. **e–g** Modified neurological severity score (mNSS), foot-fault test data and wire grip and motion test (WGMT) data showing changes in neurological function in CCI model mice with or without Drosha overexpression *(n* = 6, **P < 0.01 compared to sham mice, ##*P* < 0.01 compared to CCI model mice with Drosha overexpression). **h** Open-field test data showing changes in motor function in CCI model mice with or without Drosha overexpression (*n* = 6, **P < 0.01). The data are presented as the means ± SEMs.

Decreased Drosha expression resulting from stress is implicated in neuronal death^28,29^. In this study, we found that Drosha expression was significantly decreased in the injured cortex of mice with CCI-induced TBI. This change preceded the upregulation of Drosha mRNA and was accompanied by an increase in the expression of the apoptotic protein Bax (Fig. 1a and Supplementary Fig. 1). The expression of DGCR8, the cofactor of Drosha in miRNA biogenesis, did not change (Fig. 1a). To analyze the association between Drosha and nerve injury, we established transgenic mice overexpressing Drosha and subjected them to CCI. The results showed that overexpression of Drosha attenuated the increase in the expression of the apoptotic protein Bax in the injured cortex (Fig. 1b). Then, we evaluated cortical tissue defects by staining serial coronal sections with Nissl dye. The results indicated that Drosha overexpression significantly decreased the lesion volume in CCI model mice (Fig. 1c). Furthermore, we performed several behavioral tests to evaluate the effect of Drosha overexpression on improving neurological function of CCI model mice (Fig. 1d). The modified neurological severity score (mNSS) was used to evaluate neurological function recovery, and the results showed that functional deficits were significantly alleviated in CCI model mice with Drosha overexpression compared with CCI model mice without Drosha overexpression (Fig. 1e). The foot-fault test showed that the sensorimotor coordiation of CCI model mice with Drosha overexpression was markedly improved (Fig. 1f). In the wire grip and motion test (WGMT), we observed more pronounced recovery of forelimb muscle strength in CCI model mice with Drosha overexpression (Fig. 1g). In the open-field test, significant increases in both the distance traveled and the movement velocity were observed in CCI model mice with Drosha overexpression (Fig. 1h). Together, these data showed that Drosha expression was significantly decreased in the injured cortex in CCI model mice and that Drosha overexpression markedly alleviated nerve injury and neurological deficits.

### The decrease in Drosha expression is a result of Cdk5-mediated phosphorylation

**Fig. 2:**
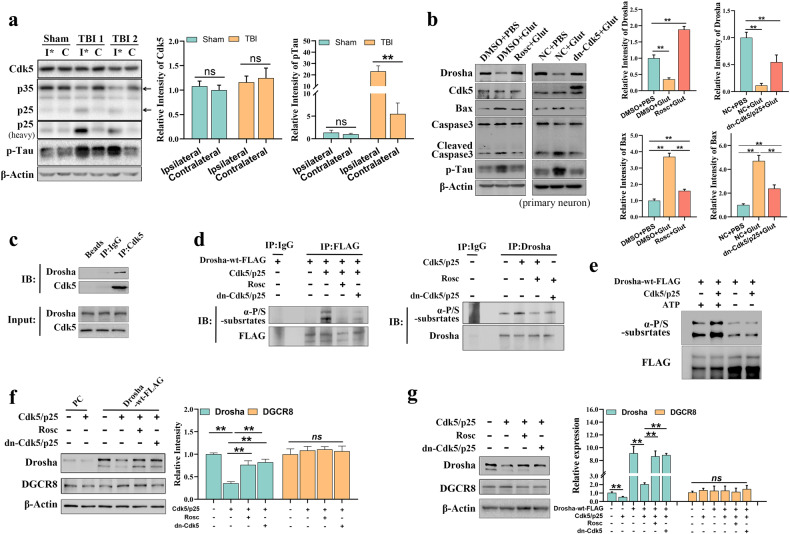
The decrease in Drosha expression is a result of Cdk5-mediated phosphorylation. **a** WB showing the changes in Cdk5, p25 and p-Tau levels in the injured (I*) and corresponding (C) cortices of CCI model mice (*n* = 4, **P < 0.01). **b** WB showing the change in the expression of Drosha and the apoptotic proteins Bax and cleaved caspase 3 in primary cultured neurons treated with glutamate and pretreated with or without Rosc or overexpressing dn-Cdk5 and p25 (*n* = 4, **P < 0.01). **c** Co-immunoprecipitation showing the protein interaction between Drosha and Cdk5. **d** WB following immunoprecipitation showing the phosphorylation of Drosha-wt-FLAG and Drosha in HEK-293T cells coexpressing Cdk5 and p25 and pretreated with or without Rosc or overexpressing both dn-Cdk5 and p25. **e** In vitro kinase assay showing the phosphorylation of purified Drosha-wt-FLAG. **f** and **g** Quantification of the protein levels of Drosha-wt-FLAG, Drosha and DGCR8 in HEK-293T cells coexpressing Cdk5 and p25 and pretreated with or without Rosc or overexpressing both dn-Cdk5 and p25 (*n* = 4, **P < 0.01). The data are presented as the means ± SEMs.

Emerging evidence indicates that excitotoxicity plays an important role in the sequential multifactorial cascade associated with TBI, and excitotoxicity causes calcium overload and results in the activation of calpain^10^. Activated calpain cleaves p35 into p25, which aberrantly activates Cdk5 and directs its activity toward a different set of substrates to induce neuronal damage^30^. Here, we found that the levels of p25 and phosphorylated Tau, a classical substrate of Cdk5, were increased in the injured cortex of CCI model mice (Fig. 2a). Treatment of primary cultured neurons with glutamate decreased Drosha expression and increased cleaved Caspase 3 and Bax expression. In contrast, these effects were attenuated by pretreatment with the CDK inhibitor roscovitine (Rosc) or the overexpression of dn-Cdk5 (Cdk5 inactive mutant), indicating that the decrease in Drosha expression was closely associated with abnormal Cdk5 activation (Fig. 2b).

A previous study suggested that the preferred sites for Ser/Thr phosphorylation by Cdk5 are within the consensus motif (S/T)PX(K/H/R)^4^. Here, we found several potential sites (S221, S255, T274, S300, S355, and T1008) for Drosha phosphorylation by Cdk5, most of which are conserved among humans, monkeys, mice and rats and are located in the N-terminus (Supplementary Fig. 2). We aimed to elucidate whether Drosha is indeed a phosphorylation substrate of Cdk5; we first demonstrated that Cdk5 specifically pulled down endogenous Drosha by coimmunoprecipitation (Fig. 2c). Then, we immunoprecipitated Drosha and probed the immunoprecipitates with an antibody that specifically recognizes phosphorylation targets of CDK (α-P/S substrates). The results showed that coexpressing Cdk5 and p25 increased the phosphorylation of overexpressed Drosha-wt-FLAG and that this change was alleviated by either Rosc pretreatment or overexpression of both dn-Cdk5 and p25 (Fig. 2d left panel). Similar data were obtained for endogenous Drosha (Fig. 2d right panel). A kinase assay further showed that Cdk5 directly phosphorylated Drosha in vitro (Fig. 2e). Finally, we confirmed the association between the protein level of Drosha and Cdk5-mediated phosphorylation in HEK-293T cells. The results showed that coexpressing Cdk5 and p25 led to a decrease in the levels of both Drosha-wt-FLAG and endogenous Drosha, but Rosc pretreatment and overexpression of both dn-Cdk5 and p25 restored the levels of Drosha-wt-FLAG and Drosha, respectively (Fig. 2f and g); this result was consistent with the effect of abnormally activated Cdk5 on Drosha expression in primary cultured neurons. We previously showed that the phosphorylation of Drosha facilitates its cleavage by calpain and subsequent degradation. To further elucidate the link between phosphorylation and decreased Drosha protein levels, we transfected Drosha-WT-FLAG, Cdk5 and p25 into HEK-293T cells and then examined Drosha phosphorylation and protein expression levels in the presence or absence of calpeptin treatment. The data showed that the calpain inhibitor calpeptin attenuated the Cdk5-mediated reduction in Drosha protein expression. Moreover, we also showed that Drosha protein levels were decreased after the coexpression of Cdk5 and p25, which has been shown to increase Drosha phosphorylation. Interestingly, inhibition of calpain promoted the accumulation of phosphorylated Drosha under conditions of Cdk5 and p25 coexpression (Supplementary Fig. 3).

**Fig. 3:**
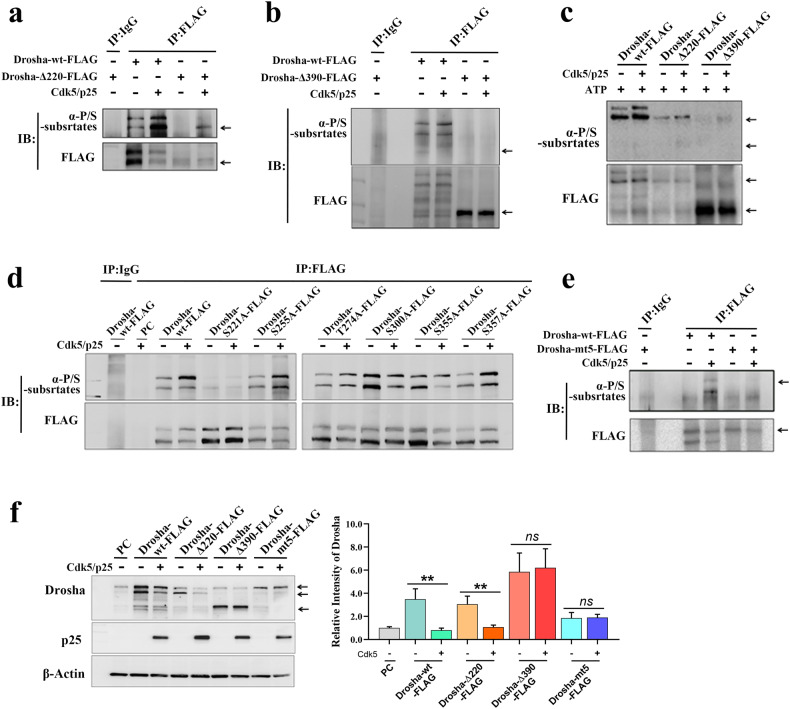
Drosha is phosphorylated at multiple sites in its RS-rich domain by Cdk5. **a** WB following immunoprecipitation showing the phosphorylation of Drosha and the Drosha truncation mutant Drosha-∆220-FLAG in HEK-293T cells expressing both Cdk5 and p25. **b** WB following immunoprecipitation showing the phosphorylation of Drosha and the Drosha truncation mutant Drosha-∆390-FLAG in HEK-293T cells expressing both Cdk5 and p25. **c** In vitro kinase assay showing the phosphorylation of purified Drosha-wt-FLAG, Drosha-∆220-FLAG and Drosha-∆390-FLAG. **d** WB following immunoprecipitation showing the phosphorylation of single-amino acid Drosha mutants in HEK-293T cells expressing both Cdk5 and p25. **e** WB following immunoprecipitation showing the phosphorylation of the multiple-amino acid Drosha mutant Drosha-mt5-FLAG in HEK-293T cells expressing both Cdk5 and p25. **f** Quantification of the protein levels of Drosha and Drosha mutants in HEK-293T cells expressing both Cdk5 and p25 or not by WB (*n* = 4, **P < 0.01). The data are presented as the means ± SEMs.

To identify the phosphorylation sites in Drosha, full-length Drosha (Drosha-wt-FLAG) and Drosha deletion mutants (Drosha-∆220-FLAG [N’-end pro-rich domain deletion mutant] and Drosha-∆390-FLAG [N’-end pro-rich domain and RS-rich domain deletion mutant]) were overexpressed. The analysis showed that both Drosha-wt-FLAG and Drosha-∆220-FLAG, but not Drosha-∆390-FLAG, were phosphorylated when Cdk5 and p25 were coexpressed (Fig. 3a and b). A kinase assay was used to further verify the results in vitro (Fig. 3c). These results suggested that Cdk5 phosphorylation sites are present in the region between amino acid residues 220 and 390, which is consistent with our previous analysis. Furthermore, to identify the phosphorylated amino acid residues, Drosha mutants with single amino acid substitutions (S221A, S255A, T274A, S300A, and S355A, in which serine/threonine was mutated to alanine) were generated. The levels of phosphorylated Drosha-S211A-FLAG, Drosha-T274A-FLAG, Drosha-S300A-FLAG and Drosha-S355A-FLAG were decreased compared with those of phosphorylated Drosha-wt-FLAG (Fig. 3d). However, a mutant in which multiple amino acid were altered (Drosha-mt5-FLAG [S220A, S255A, T274A, S300A, and S355A]) was phosphorylated at a lower level (Fig. 3e). To determine the role of phosphorylation in Drosha stabilization, we measured the levels of Drosha-wt-FLAG, Drosha-∆220-FLAG, Drosha-∆390-FLAG, and Drosha-mt5-FLAG when Cdk5 and p25 were overexpressed. The results showed that the levels of Drosha-wt-FLAG and Drosha-∆220-FLAG were markedly decreased compared with those of Drosha-∆390-FLAG and Drosha-mt5-FLAG (Fig. 3f).

Together, these results suggested that Drosha was directly phosphorylated at multiple sites in its arginine/serine (RS)-rich domain by aberrantly activated Cdk5 and that the phosphorylation directly decreased Drosha levels.

### Stabilization of Drosha levels alleviates nerve injury in CCI model mice

**Fig. 4:**
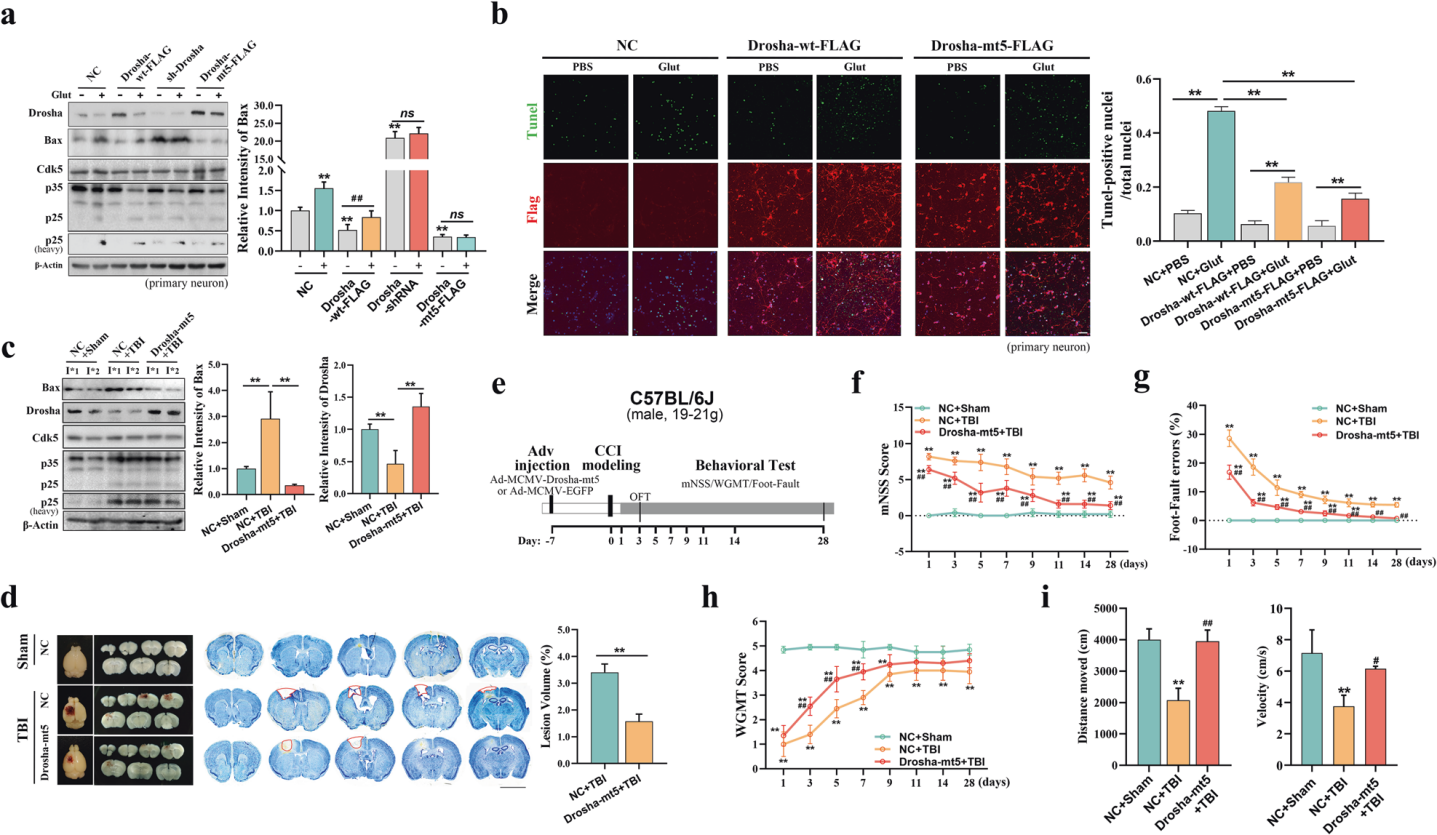
Stabilization of Drosha levels alleviates nerve injury in CCI model mice. **a** WB showing the change in the expression of the apoptotic protein Bax in glutamate-treated primary cultured neurons with Drosha-wt-FLAG overexpression, Drosha knockdown (sh-Drosha) and Drosha-mt5-FLAG overexpression (*n* = 4, **P < 0.01 compared to the NC group, ^##^*P* < 0.01). **b** TUNEL staining showing the change in apoptosis in glutamate-treated primary cultured neurons with Drosha-wt-FLAG or Drosha-mt5-FLAG overexpression (*n* = 3, **P < 0.01, bar = 100 μm). **c** WB showing the change in the expression of the apoptotic protein Bax in the injured cortex (I*) of CCI model mice with or without Drosha-mt5 overexpression (numbers 1 and 2 represent the different mice in the group, *n* = 4, **P < 0.01). **d** Left: Morphological analysis of cortical lesions in CCI model mice with or without Drosha-mt5 overexpression. Middle: Images of Nissl staining showing the lesion volume in the cortex of CCI model mice with or without Drosha-mt5 overexpression. Right: Statistical analysis of the cortical lesion volume in CCI model mice with or without Drosha overexpression (*n* = 4, **P < 0.01, bar = 4 mm). **e** Schematic showing the protocol used to generate Drosha-mt5-overexpressing mice and the experimental timelines. **f**–**h** mNSS, foot fault test data and WGMT data showing changes in neurological function in CCI model mice with or without Drosha-mt5 overexpression (*n* = 6, **P < 0.01 compared to the sham group, #*#P* < 0.01 compared to CCI model mice without Drosha-mt5 overexpression). **i** Open-field test data showing changes in motor function in CCI model mice with or without Drosha-mt5 overexpression (*n* = 6, **P < 0.01 compared to the sham group, ^##^*P* < 0.01 compared to CCI model mice without Drosha-mt5 overexpression). The data are presented as the means ± SEMs.

We revealed that in primary cultured neurons, Drosha-mt5-FLAG overexpression could significantly inhibit the increase in Bax expression under excitatory stress and that, conversely, knockdown of Drosha exerted the opposite effect (Fig. 4a). TUNEL staining confirmed that Drosha-mt5-FLAG overexpression alleviated glutamate-induced neuronal apoptosis (Fig. 4b).

To verify the association between Drosha stability and nerve injury in CCI model mice, Drosha-mt5 was overexpressed by the stereotactic injection of adenovirus into the primary motor cortex (M1/M2 region). The mice were subjected to CCI in the same region after Drosha-mt5 overexpression. Nerve injury was assessed, and the data showed that overexpression of Drosha-mt5 suppressed the increase in the expression of the apoptotic protein Bax in the injured cortex (Fig. 4c and Supplementary Fig. 4a). Nissl staining revealed that Drosha-mt5 overexpression significantly decreased the cortical lesion size in CCI model mice (Fig. 4d and Supplementary Fig. 4c). We further evaluated the protective effect of Drosha-mt5 overexpression on neurological function (Fig. 4e and Supplementary Fig. 4b). In CCI model mice overexpressing Drosha-mt5, neurological function was restored, as measured by the mNSS (Fig. 4f and Supplementary Fig. 4d), and sensorimotor coordination deficits were significantly attenuated, as determined by the foot-fault test (Fig. 4g). CCI model mice overexpressing Drosha-mt5 exhibited better forelimb muscle strength in the WGMT (Fig. 4h and Supplementary Fig. 4e) and marked improvement of motor function in the open-field test (Fig. 4i and Supplementary Fig. 4f).

### Drosha is an effective target for early intervention in TBI

**Fig. 5:**
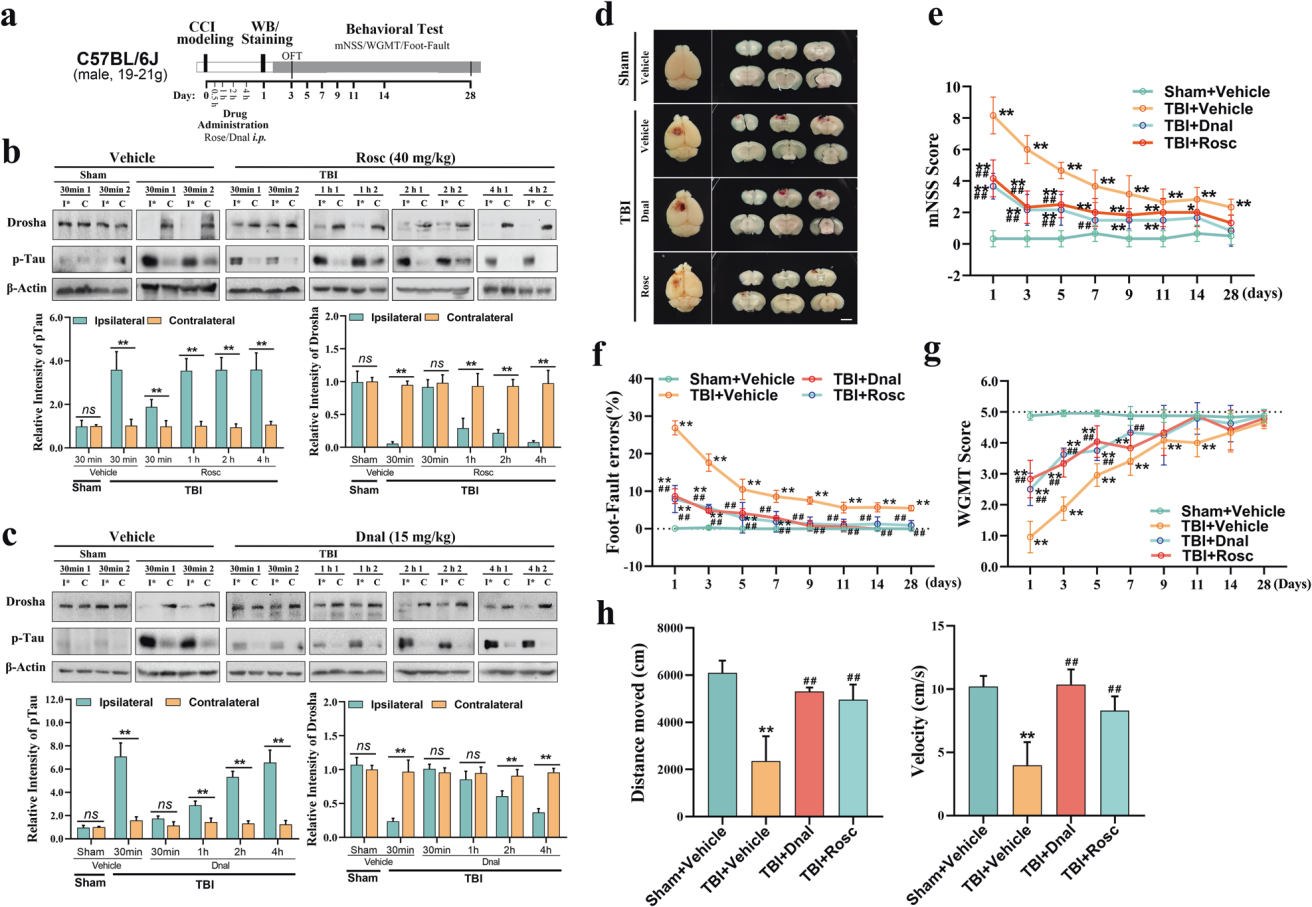
Drosha is an effective target for the early treatment of TBI. **a** Schematic showing the timeline of drug administration, morphological analysis, and behavioral testing of CCI model mice. **b** WB showing the stability of Drosha expression over time following the intraperitoneal injection of Rosc after CCI (*n* = 6, **P < 0.01). **c** WB showing the stability of Drosha expression over time following the intraperitoneal injection of Dnal after CCI (*n* = 6, **P < 0.01). **d** Morphological analysis of tissue lesions in CCI model mice after Rosc (30 min) or Dnal (1 h) treatment after CCI (bar = 4 mm). **e**–**g** mNSS, foot-fault test data and WGMT data showing changes in neurological function in CCI model mice treated with Rosc (30 min) or Dnal (1 h) after CCI (*n* = 6, **P < 0.01 compared to vehicle-injected sham mice, ^##^*P* < 0.01 compared to the vehicle-injected TBI group). **h** Open-field test data showing changes in motor function in CCI model mice treated with Rosc (30 min) or Dnal (1 h) after CCI (*n* = 6, **P < 0.01 compared to the vehicle-treated sham group, ^##^*P* < 0.01 compared to the vehicle-injected TBI group). The data are presented as the means ± SEMs.

The unpredictability of TBI progression and the short therapeutic window limit the early administration of neuroprotective treatment for TBI. We demonstrated that maintaining Drosha levels exerts neuroprotective effects in CCI model mice. Considering that pan-CDK inhibitors are frequently used in tumor treatment and that some of them have a markedly high affinity for Cdk5, indirectly regulating the stability of Drosha expression by inhibiting Cdk5 seems to be a feasible and cost-effective strategy for achieving neuroprotective effects early after TBI. Here, we selected two inhibitors of CDKs (roscovitine (Rosc) and dinaciclib (Dnal)), which are currently in clinical trials for cancer treatment, due to their high specificity for Cdk5 and low inhibitory concentration^31^, and we evaluated their ability to modulate Drosha expression in TBI models (Fig. 5a). To determine the time frame for maintaining Drosha expression, Rosc and Dnal were intraperitoneally injected at different time points ranging from 30 min to 4 h after CCI. The analysis showed that administration of Rosc 30 min after CCI and Dnal 1 h after CCI maintained Drosha levels in the injured cortex (Fig. 5b and c). Morphological analysis of serial coronal sections revealed that the lesion size in CCI model mice was significantly decreased by the administration of Rosc (30 min) and Dnal (1 h); therefore, the time frame for alleviating tissue lesions was consistent with that for maintaining Drosha levels (Fig. 5d).

As mentioned in the previous sections, we hypothesized that the stabilization of Drosha levels exerts neuroprotective effects after TBI. Therefore, a series of behavioral tests were performed to evaluate the effect of stabilizing Drosha expression on neurological function in CCI model mice. According to the mNSS, treatment with Rosc (30 min) and Dnal (1 h) promoted the recovery of neurological function (Fig. 5e). In the foot-fault test, CCI model mice treated with Rosc (30 min) and Dnal (1 h) exhibited significantly improved sensorimotor coordination (Fig. 5f). Forelimb muscle strength and motor function were significantly improved in CCI model mice after Rosc (30 min) and Dnal (1 h) treatment, as shown in the wire grip and motion test (Fig. 5g) and the open-field test (Fig. 5h), respectively. These results revealed that CDK inhibitors have great potential for stabilizing Drosha levels and ameliorating neurological function in CCI model mice.

### Drosha is an indicator of the effectiveness of CDK inhibitors in early treatment after TBI

**Fig. 6:**
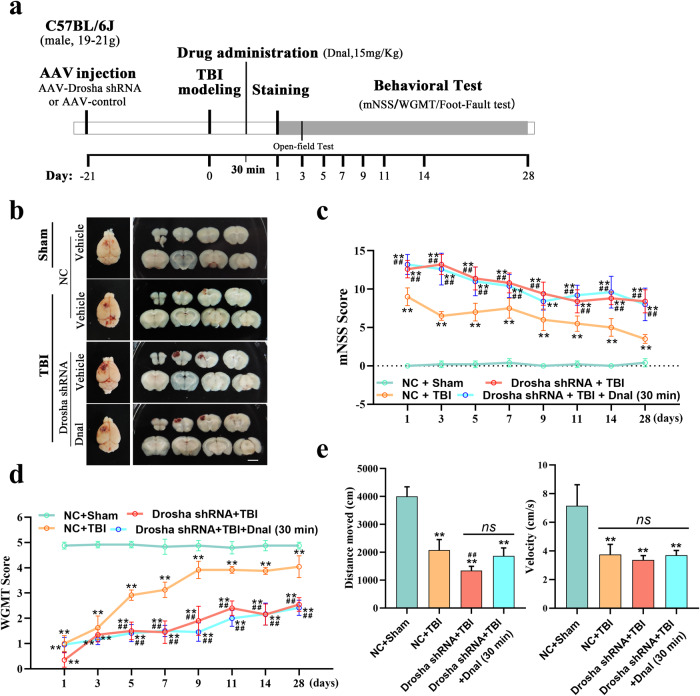
Drosha is an indicator of the effectiveness of CDK inhibitors in the early treatment of TBI. **a** Schematic showing the timeline of AAV-Drosha shRNA injection, drug administration, morphological analysis, and behavioral testing of CCI model mice. **b** Morphological analysis of brain lesions in CCI model mice treated with Dnal with or without Drosha knockdown (bar = 3 mm). **c** and **d** mNSS and WGMT data showing changes in neurological function in Dnal treated CCI model mice with or without Drosha knockdown (*n* = 6, **P < 0.01 compared to the vehicle-injected sham group, ^##^*P* < 0.01 compared to the vehicle-injected TBI group). **e** Open-field test data showing changes in motor function in Dnal-treated CCI model mice with or without Drosha knockdown (*n* = 6, **P < 0.01 compared to the vehicle-injected sham group, ^##^*P* < 0.01 compared to the vehicle-injected TBI group). The data are presented as the means ± SEMs.

CDK inhibitors showed great therapeutic potential for early treatment after TBI. However, since Cdk5 participates in nerve injury by targeting multiple substrates, we further assessed whether Drosha is the key target of Cdk5 and whether it could be an indicator of the effectiveness of CDK inhibitors in the treatment of TBI. We knocked down Drosha in the primary motor cortex (M1/M2 region) before CCI. Nerve injury was measured as shown in the schematic in Fig. 6a. The neuroprotective effects of CDK inhibitors in CCI model mice with Drosha knockdown were evaluated by analyzing serial coronal sections. The results showed that the CDK inhibitor Dnal failed to alleviate nerve injury in CCI model mice with Drosha knockdown (Fig. 6b). According to the mNSS, Drosha knockdown exacerbated neurological impairment, and Dnal did not promote neurological function recovery (Fig. 6c). Dnal had no effect on the motor function of CCI model mice with Drosha knockdown in the WGMT or open-field test (Fig. 6d and e). Together, these results showed that the neuroprotective effect of CDK inhibitors in TBI is mediated by Drosha and that Drosha is an indicator of the effectiveness of CDK inhibitors in early treatment after TBI.

## Discussion

TBI causes neurological deficits through a pathological multifactorial cascade that involves and is promoted by kinases. Studies have demonstrated that the kinase Cdk5 is abnormally activated under multiple stress conditions^32^, and some researchers have shown that inhibiting Cdk5 activity exerts neuroprotective effects on stroke, TBI and neurodegenerative diseases^33^. However, the key substrate of Cdk5 varies in different diseases. In this study, we found that the expression of Drosha, a enzyme that mediates miRNA biogenesis, was reduced in the injured cortex in TBI and that this change was accompanied by Cdk5 overactivity. We demonstrated that the decrease in Drosha expression was the result of Cdk5-mediated phosphorylation.

Drosha contains two RNase III domains (RIIIDa and RIIIDb), a dsRNA-binding domain (dsRBD) in the C-terminus, a central domain in the middle and a proline-rich and RS-rich domain in the N-terminus^34^. We demonstrated in vitro and in vivo that the five specific sites that are phosphorylated by Cdk5 are located in the RS-rich domain of Drosha. The RS-rich domain is not needed for pri-miRNA processing but is essential for the proper subcellular localization of Drosha. We found that Drosha was widely distributed in the nucleus and cytoplasm in primary cultured cortical neurons and that Drosha expression in both compartments was significantly decreased under excitotoxic conditions (Supplementary Fig. 5). As recent research has revealed the existence of cytoplasmic Drosha and its noncanonical function^35^, our data suggest that Drosha may play both canonical and noncanonical roles in neurons.

Drosha deficiency affects cell function and even cell fate^36,37^. Our previous studies have shown that stress-induced Drosha loss contributes to neuronal toxicity in neurodegenerative diseases such as PD and AD^28,38^. In this study, we showed that Drosha expression was decreased in the injured cortex and that overexpressing Drosha mitigated miRNAs dysregulation (Supplementary Fig. 6), alleviated neuronal damage, reduced the degree of nerve injury, and ameliorated neurological deficits in CCI mice. TBI is characterized by sudden onset and rapid progression, which limits the effectiveness of early neuroprotective treatments after trauma. Molecules that are both neuroprotective and easily administered on-scene are beneficial for the early treatment of TBI. Recently, Cdk5 was proposed to play a vital role in cancer development, and numerous high-affinity inhibitors of Cdk5 have been designed^39^. Roscovitine, whose safety has been proven in clinical trials for cancer treatment, has been used for the treatment of neurological emergencies such as stroke and TBI in various preclinical studies^40^. Many studies on the neuroprotective effect of roscovitine have revealed that it inhibits Cdk5 activity. However, since roscovitine is a pan-CDK inhibitor, its precise targets and downstream pathway remain to be elucidated. Furthermore, in most previous studies, roscovitine was administered before trauma, which is not conducive to treating TBI with unpredictable onset. In this study, we confirmed the neuroprotective effect of roscovitine on TBI, revealed that the Cdk5-Drosha regulatory pathway is an effective target for neuroprotective strategies after TBI, and identified the role of Drosha in related therapeutic strategies. Importantly, we showed that the optimal therapeutic time frame for the intraperitoneal injection of roscovitine was within 30 min post-injury and that this drug has great potential for the emergency treatment of TBI.

Drug repurposing, redirecting, and repositioning, which involve finding new uses for existing drugs that have entered clinical trials and shown good inhibitory effects and few side effects, have recently become less expensive alternatives to drug development^41,42^. In this study, we identified dinaciclib as another candidate for the early treatment of TBI based on its ability to regulate the Cdk5-Drosha pathway and thus exert neuroprotective effects. Dinaciclib, also known as MK-7965 or SCH727965, is currently the most promising Cdk5 inhibitor, as it has a stronger inhibitory effect and higher selectivity for Cdk5 than other Cdk5 inhibitors^43^. Its side effects have been identified, and its safety has been verified in clinical trials for the treatment of various cancers^44^. In this study, we demonstrated that administration of dinaciclib 1 h pos-tinjury could maintain Drosha levels and attenuate nerve injury in CCI mice.

In summary, we identified Drosha, a key miRNA-related enzyme, as an important target of Cdk5 for the first time, and we revealed that inhibiting the Cdk5-Drosha regulation exerts neuroprotective effects after TBI. Following the principle of drug repurposing, we revealed the clinical potential of Cdk5 inhibitors for the early treatment of TBI. Although this study could be improved, the current work is sufficient to provide new ideas for the development of drugs for the clinical treatment of TBI and highlights the clinical potential of targeting the Cdk5-Drosha regulation early after TBI to achieve neuroprotection.

### Supplementary information

SUPPLEMENTARY MATERIALS
