# Supplementary material for: Maintaining Drosha expression with Cdk5 inhibitors as a potential therapeutic strategy for early intervention after TBI
Source: Exp Mol Med. 2024 Jan 10;56(1):210–9. doi: 10.1038/s12276-023-01152-4 (PMC10834983; doi:10.1038/s12276-023-01152-4)
Supplement: Supplementary file 1 — SUPPLEMENTARY MATERIALS [file 12276_2023_1152_MOESM1_ESM.pdf]

SUPPLEMENTARY MATERIALS

Maintaining Drosha expression with Cdk5 inhibitors as a potential therapeutic strategy for early intervention after TBI

Lu Huang[a,b†], Li Xia[a,c†], Tiejian Nie[a], Bozhou Cui[a], Jianjun Lu[a], Fangfang Lu[a], Feiyan Fan[a], Dongni Ren[a], Yuan Lu[a], Guodong Gao[c], Qian Yang[a*]

[a] Department of Experimental Surgery, Tangdu Hospital, The Fourth Military Medical University, Xi'an 710038, Shaanxi, China.

[b] Department of Anesthesiology, Tangdu Hospital, The Fourth Military Medical University, Xi'an 710038, Shaanxi, China.

[c] Department of Neurosurgery, Tangdu Hospital, The Fourth Military Medical University, Xi'an 710038, Shaanxi, China.

[*] Corresponding author is Qian Yang. Email: qianyang@fmmu.edu.cn.

[†] These authors contributed equally to this work.

Supplementary Figures

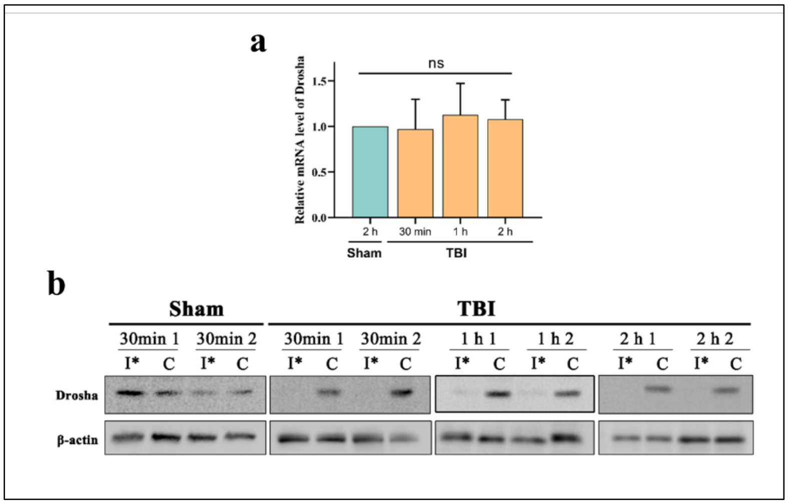

Supplementary Fig. 1: The stability of Drosha in TBI mice.

(a) RT-qPCR showing the timeline of Drosha mRNA level after CCI modeling (n = 6, **P < 0.01 compared to sham group). **(b)** Representative WB showing the timeline of Drosha stability maintenance after CCI modeling.

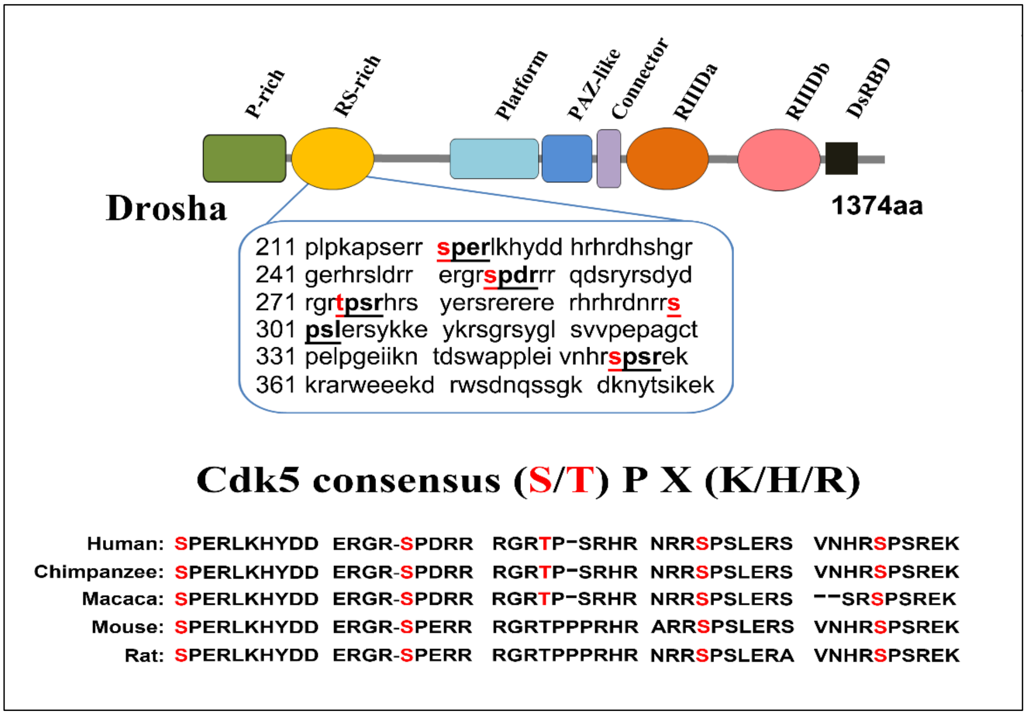

Supplementary Fig. 2: Potential phosphorylation sites for Cdk5 on Drosha

Several potential phosphorylation sites for Cdk5 (S221, S255, T274, S300, S355, T1008) were located on Drosha (the amino acid sequence was obtained from UniProtKB - Q9NRR4). The conservation of the sites at the N-terminus was shown across species in Human, Chimpanzee Macaca, Mouse, and Rat schematically.

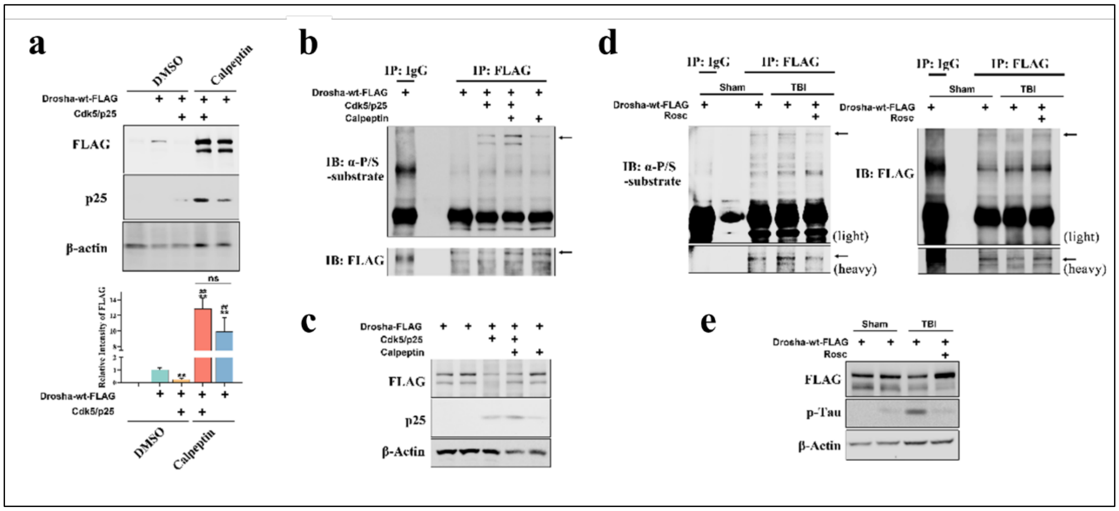

Supplementary Fig. 3: Drosha decline is a result of Cdk5-mediated phosphorylation.

(a) Quantification of the protein level of Drosha-wt-FLAG in HEK-293T with different treatment by WB (n=4, **P < 0.01 compared to Drosha-wt-Flag overexpression with DMSO treatment, ##P < 0.01 compared to Drosha-wt-Flag, Cdk5 and p25 overexpression with DMSO treatment). **(b)** WB following immunoprecipitation showing the phosphorylation of Drosha-wt-FLAG in HEK-293T cells coexpressing Cdk5 and p25 and treated with or without calpeptin. **(c)** Representative WB showing the protein level of Drosha-wt-FLAG with different treatment in HEK-293T whole cell lysates. **(d)** WB following immunoprecipitation showing the phosphorylation of Drosha-wt-FLAG in TBI mice injured brain tissue with or without Rosc post-treatment. **(e)** Representative WB showing the protein level of Drosha-wt-FLAG with different treatment in whole brain tissue lysates of TBI mice.

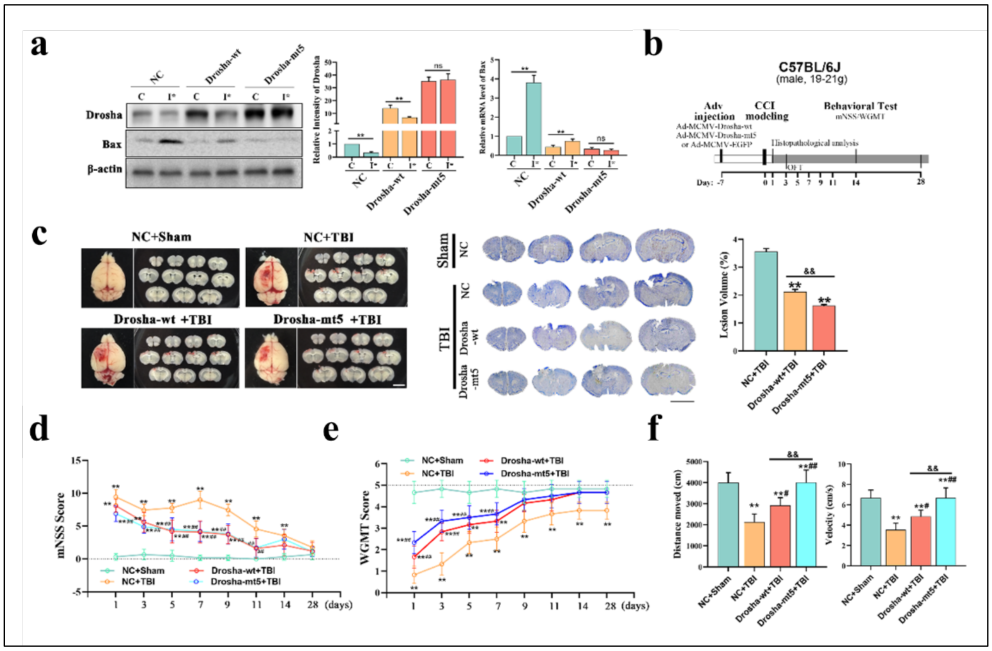

Supplementary Fig. 4: Comparison of neuroprotective effects of Drosha-wt and Drosha-mt5 overexpression.

(a) WB showing the change of Drosha-wt, Drosha-mt5 and apoptotic protein Bax in the injured brain tissue in TBI mice with Drosha-wt or Drosha-mt5 pre-expression (n=4, $**P < 0.01$). **(b)** Schematic showing the timeline of Adv injection, morphological assay, and behavioral test of TBI mice. **(c)** Left: The morphology showing the tissue defect in TBI mice with Drosha-wt or Drosha-mt5 pre-expression; Middle: Nissl staining showing the tissue lesion in the TBI mice with Drosha-wt or Drosha-mt5 pre-expression; Right: Statistical results showing the cortical lesion volume of TBI mice with Drosha-wt or Drosha-mt5 pre-expression. (n = 4, $**P < 0.01$ compared to sham group, $^{\&\&}P < 0.01$, bar = 4 mm). **(d-f)** The mNSS, the WGMT and the open-field test showing the neurological function changes of TBI mice with Drosha-wt or Drosha-mt5 pre-expression. (n = 6, $**P < 0.01$ compared to Sham, $\#\#P < 0.01$ compared to TBI group, $^{\&\&}P < 0.01$).

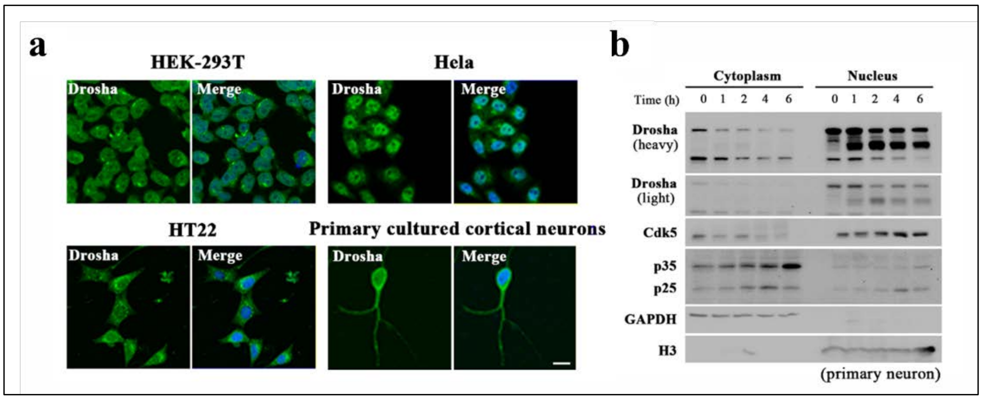

Supplementary Fig. 5: The distribution of Drosha in different type of cells.

(a) Immunofluorescence showed the variation of Drosha distribution in different cell lines (bar = 30 μm). **(b)** Representative WB showing the change of cytoplasmic and nuclear Drosha in the primary cultured cortical neurons under the treatment of glutamate.

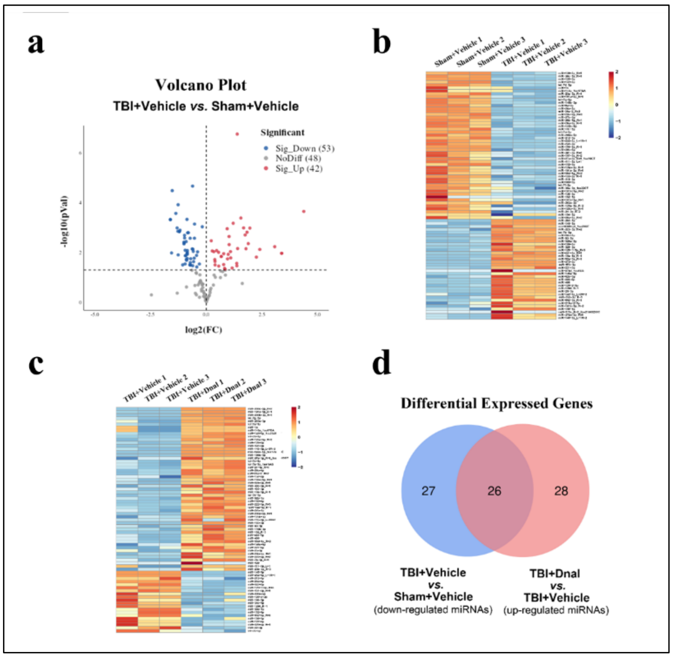

Supplementary Fig. 6: miRNA sequencing analysis of TBI mice with Dnal treatment

(a) Volcano map shows the changed miRNAs in TBI mice with vehicle treatment (TBI+vehicle group) compared with Sham mice with vehicle treatment (Sham+vehicle group). **(b)** Heat map shows the changed miRNAs in TBI mice with vehicle treatment (TBI+vehicle group) compared with Sham mice with vehicle treatment (Sham+vehicle group). **(c)** Heat map shows the changed miRNAs in TBI mice with Dnal treatment (TBI+Dnal group) compared with TBI mice with vehicle treatment (TBI+vehicle group). **(d)** Venn analysis showing the intersection of miRNAs that changes in each group.

Supplementary Tables

Gene	Forward (5' to 3')	Reverse (5' to 3')
S221A	CAGCCTTTCTGGGGCCCTTCTCTCACTGG	CCAGTGAGAGAAGGGCCCCAGAAAGGCTG
S255A	TCTCCTGTCGGGAGCGCGGCCTCGCTCC	GGAGCGAGGCCGCGCTCCCGACAGGAGA
T274A	GTGGCGAGATGGTGCTCTCCCTCGGTCAT	ATGACCGAGGGAGAGCACCATCTCGCCAC
S300A	CCTTTCCAGAGATGGTGCTCTTCGGTTGTCTCGAT	ATCGAGACAACCGAAGAGCACCATCTCTGGAAAGG
S355A	CTTCTCCCTACTTGGGGCGCGATGATTCACAATCT	AGATTGTGAATCATCGCGCCCCAAGTAGGGAGAAG

Supplementary Table 1: Primers used for Drosha mutant plasmid preparation.
